# Bioactive proteins and peptides isolated from Chinese medicines with pharmaceutical potential

**DOI:** 10.1186/1749-8546-9-19

**Published:** 2014-07-19

**Authors:** Kam Lok Wong, Ricky Ngok Shun Wong, Liang Zhang, Wing Keung Liu, Tzi Bun NG, Pang Chui Shaw, Philip Chi Lip Kwok, Yau Ming Lai, Zhang Jin Zhang, Yanbo Zhang, Yao Tong, Ho-Pan Cheung, Jia Lu, Stephen Cho Wing Sze

**Affiliations:** 1 School of Chinese Medicine, LKS Faculty of Medicine, The University of Hong Kong, 10 Sassoon Road, Pokfulam, Hong Kong Special Administrative Region, China; 2 Department of Biology, Faculty of Science, Hong Kong Baptist University, Hong Kong Special Administrative Region, China; 3 School of Biomedical Sciences, Faculty of Medicine, The Chinese University of Hong Kong, Shatin, N.T., Hong Kong Special Administrative Region, China; 4 School of Life Sciences and Centre for Protein Science and Crystallography, Faculty of Science, The Chinese University of Hong Kong, Shatin, N.T., Hong Kong Special Administrative Region, China; 5 Department of Pharmacology & Pharmacy, LKS Faculty of Medicine, The University of Hong Kong, Pokfulam, Hong Kong Special Administrative Region, China; 6 Department of Health Technology and Informatics, Hong Kong Polytechnic University, Hung Hom, Hong Kong Special Administrative Region, China

## Abstract

Some protein pharmaceuticals from Chinese medicine have been developed to treat cardiovascular diseases, genetic diseases, and cancer. Bioactive proteins with various pharmacological properties have been successfully isolated from animals such as *Hirudo medicinalis* (medicinal leech), *Eisenia fetida* (earthworm), and *Mesobuthus martensii* (Chinese scorpion), and from herbal medicines derived from species such as *Cordyceps militaris*, *Ganoderma, Momordica cochinchinensis*, *Viscum album*, *Poria cocos*, *Senna obtusifolia*, *Panax notoginseng*, *Smilax glabra*, *Ginkgo biloba*, *Dioscorea batatas*, and *Trichosanthes kirilowii*. This article reviews the isolation methods, molecular characteristics, bioactivities, pharmacological properties, and potential uses of bioactive proteins originating from these Chinese medicines.

## Background

The therapeutic potential of proteins from Chinese medicine (CM) has not realized without extensive research. Nevertheless, according to a report from BCC Research LLC. in October 2013, the global market for protein pharmaceuticals was predicted to be $136.7 billion in 2013 and to increase to $179.1 billion in 2018, with an average annual growth rate of approximately 5.6% from 2013 to 2018 [1]. Many protein pharmaceuticals are available for treating rheumatoid arthritis, coronary artery thrombosis, multiple sclerosis, and chronic lymphocytic leukemia [2-4].

Quality control of Chinese medicinal herbs is a challenge because the therapeutic effects of medicinal herbs are subject to by different factors, such as geographical constraints, soil mineral content, temperature, and humidity. Moreover, guidelines for quality control are not readily available. The 2002 guidelines of the European Agency for the Evaluation of Medicinal Products for good agricultural practice does not include a standardization for materials of herbal origin [5]. However, protein or peptide pharmaceuticals derived from CM can be produced through recombinant technology, which can minimize batch-to-batch variations in quality [6]. Additionally, drug delivery systems, such as polyethylene glycol (PEG) and nanocarriers, could promote the clinical efficacy of protein drugs from CM [7,8].

This article aims to review the bioactive proteins and peptides isolated from CM with potential for clinical use.

### Methodology

A variety of databases, including Google Scholar/Google, PubMed, Science direct, CINAHL Plus, Cochrane Library, Global health, ISI Web of Knowledge, Chinese database CNKI, CQVIP, and CJFD, were searched to get the information about protein drugs derived from Chinese medicine with the following key words: “Chinese medicine”, “natural products” together with “protein”, “peptide”, “protein pharmaceuticals”, “peptide pharmaceuticals” or “protein drugs”. Furthermore, information on the website of WHO was also collected. For the bioinformatics information of protein, the protein structure, sequence alignment or both, were obtained from the Molecular Modeling Database (MMDB) and Conserved Domain Database (CDD) on the website of NCBI by searching their scientific names of species and their protein names. All data were further analyzed to obtain the information about the current progress in research on promising protein/peptide drugs isolated from CM.

### Bioactive proteins and peptides from Chinese medicines

Raw and fresh CM materials, rather than dried, processed, or powdered materials, are often used to obtain bioactive peptides to prevent protein denaturation and degradation from processing [9,10]. Some isolated proteins have been approved by the Food and Drug Administration (FDA) of the United States for clinical use or clinical trials (Table 1).

**Table 1:** Bioactive proteins isolated from Chinese medicine and their reported pharmacological effects

**CM**	**Pharmacologically active proteins or peptides**	**Pharmacological effects**
**Peptides/protein from medicinal animals**		
*Hirudo spp.* (Leeches)	Hirudin	Anti-coagulation activity through inhibition of thrombin activity [13,14]
		Anti-proliferative activity toward human ovarian and tongues squamous cancer cells [24-27]
*Eisenia fetida* (Earthworm)	Earthworm fibrinolytic enzyme	Anti-cancer activity against hepatoma *in vitro* and *in vivo*[33]
*Mesobuthus martensii* (Chinese scorpion)	Anti-epilepsy protein (8.3 kDa)	Anti-epilepsy activity in mice *in vivo*[36,37]
**Peptides/Protein from medicinal fungi**		
*Cordyceps militaris*	Lectin designated as CML (31 kDa)	Haemagglutinating activity in mouse and rat erythrocytes
		Mitogenic activity on mouse splenocytes [43]
	Cordymin (10.9 kDa)	Anti-fungal activity through inhibition of mycelial growth *in vitro*[44]
		Inhibitory effect on HIV-1 reverse transcriptase *in vitro*[42]
		Anti-cancer activity against MCF-7 breast cancer cells *in vitro*[42]
	Protease designated CMP (12 kDa)	Anti-fungal activity *in vitro*[44]
		Anti-cancer activity against MCF-7 breast cancer cells and bladder cancer 5637 cells *in vitro*[44]
*Ganoderma spp.* (*Ling Zhi*)	Ling Zhi-8 (12.4 kDa protein)	Immuno-modulatory activity through inducing maturation of human monocyte-derived dendritic cells and stimulating IL2 and IFN-γ secretion from CD4^+^ and CD8^+^ T cells [52], [53]
		Anti-cancer activity against lung carcinoma cell growth *in vitro* and *in vivo*[55,56]
	Lectin (a 18-kDa protein)	Haemagglutinating activity and mitogenic activity towards BALB/c mouse splenocytes [49]
		Anti-cancer activity against leukemia (L1210 and M1) cells and hepatoma (HepG2) cells [54]
*Poria cocos* (Schw.) Wolf	*P. cocos* immunomodulatory protein (35.6 kDa) (PCP)	Immunomodulatory activity through activating mouse peritoneal macrophages (RAW 264.7) [60]
**Peptides/proteins from medicinal herbs**		
*Viola tricolor*	Cyclotides	Anti-cancer activity against human cancer cells U251, MDA-MB-231, A549, DU145 and BEL-7402 *in vitro*[63]
*Momordica cochinchinensis* seeds (*Mubiezhi*)	Cochinin B (28 kDa ribosome inactivating protein)	Anti-cancer activity against human cervical epithelial carcinoma (HeLa), human embryonic kidney (HEK293) and human small cell lung cancer (NCI-H187) cell lines *in vitro*[69]
	MCoCC-1 (a 33 amino acid long peptide)	Anti-cancer activity against human melanoma cell line (MM96L) *in vitro*[67,71]
	Chymotrypsin inhibitor designated as MCoCI (7.5 kDa)	Anti-oxidative activity through activation of glutathione-S-transferase and superoxide dismutase *in vitro*[71]
		Immunomodulatory activity through stimulating the proliferation of mouse splenocytes, splenic lymphocytes, bone marrow cells and macrophages *in vitro*[67]
*Viscum album* (Chinese mistletoe)	Lectin designated as CM-1 (55 kDa)	Anti-cancer activity against CLY colon cancer cells and HT-29 colorectal cancer cells *in vivo* and *in vitro* through down-regulation of micro-RNA miR-135a & b expression [73]
	Lectin designated as ACML-55	Immunomodulatory activity through enhancing both antigen specific activation and proliferation of CD4+ and CD8+ T cells as well as number of tumor antigen specific CD8+ T cells [74]
The seeds of *Senna obtusifolia*	Novel protein (19.7 kDa)	Inhibitory effect on cholesterol biosynthesis in Chinese hamster oocytes *in vitro*[78]
*Narcissus tazetta* var. *chinenesis*	*Narcissus tazetta* lectin (26 kDa)	Antiviral activity against human syncytial virus (RSV), influenza A and influenza B viruses [80]
		Immunopotentiating activity through inducing gene expression of IL-1βand TNF-α in splenocytes and macrophages *in vivo*[81]
*Smilax glabra* rhizomes (*Tufuling*)	Smilaxin (30 kDa)	Immuno-stimulating activity *in vitro*[83]
		Anti-cancer activity against MBL2 and PU5 cells *in vitro*[83]
		Inhibitory effect on HIV-1 reverse transcriptase *in vitro*[83]
*Ginkgo biloba* seeds (*Yinxing*)	Ginkbilobin (13 kDa)	Anti-fungal activity against *Botrytis cinerea*, *Mycosphaerella arachidicola*, *Fusarium oxysporum*, *Rhizoctonia solani*, and *Coprinus comatus*[85]
		Antibacterial activity against *Staphylococcus aureus*, *Pseudomonas aeruginosa*, and *Escherichia coli*[85]
		Inhibitory effect on HIV-1 reverse transcriptase [85]
		Immuno-modulatory activity through inhibiting proliferation of murine splenocytes [85]
*Dioscorea batata*	Dioscorin (32 kDa)	Carbonic anhydrase activities [90]
		Trypsin inhibitory activities [90]
		Potential airway protective effects on A549 human airway epithelium cells [91]
		Anti-oxidant properties reflected from DPPH and hydroxyl radicals scavenging effects [93]
		Immuno-modulatory activity *in vitro* and *in vivo*[94,95]
*Trichosanthes kirilowii*	Trichosanthin (247 amino acid long peptide)	Anti-HIV activity through inhibition of serum HIV-1 p24 antigen levels and increase CD4^+^ T cell count in HIV-1 infected patients [108,109]
		Antiviral activity against hepatitis B virus [110,111]

### Animal proteins and peptides

#### Hirudin from *Hirudo* spp. (leech)

**Figure 1:**
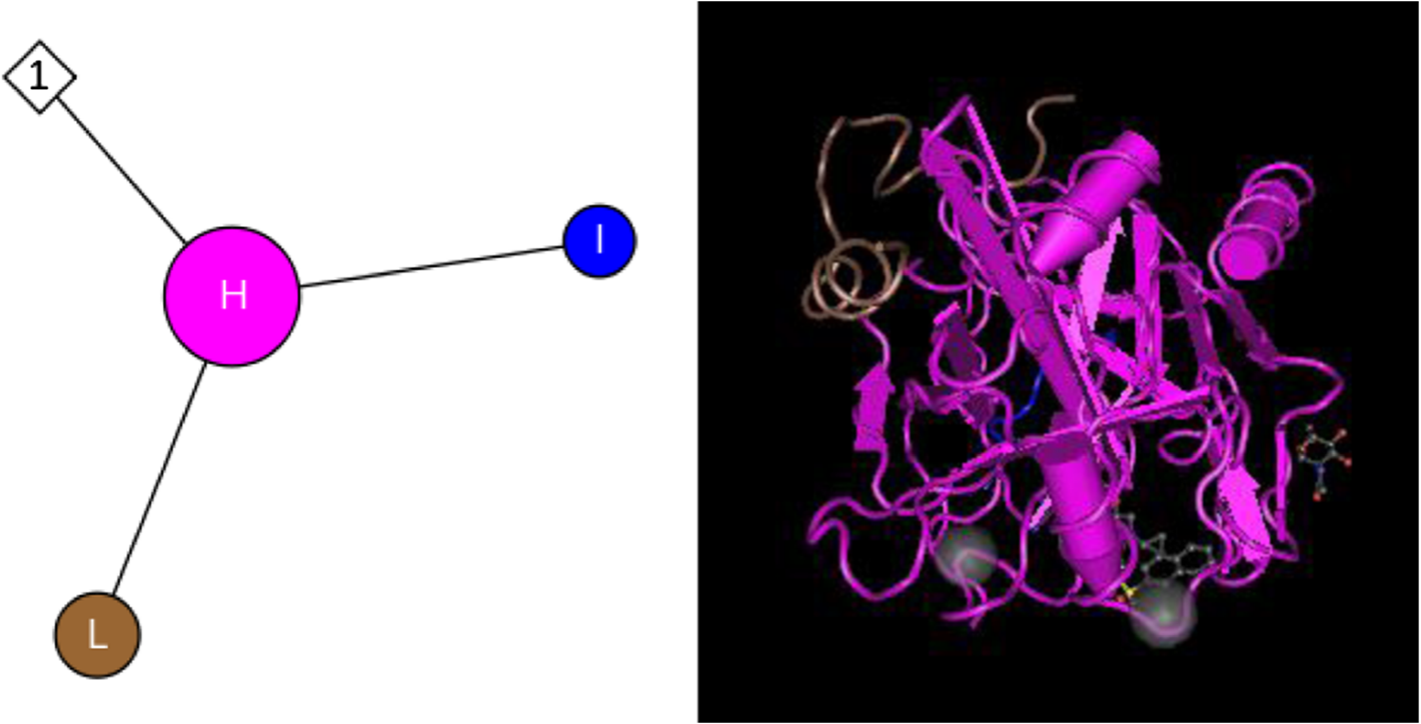
**Molecules and interactions of hirudin.** H, chain H of human thrombin; I, hirudin variant-1; L, chain L of Human thrombin. 1, Napsagatran.

Hirudin, from the saliva of *Hirudo* spp., was used in CM to enhance *blood* (*xue*) circulation [11]. Hirudin was a potent natural inhibitor of thrombin [12]*via* formation of a hirudin-thrombin complex [13,14]. Although hirudin was isolated and purified from a complex salt-containing solution with hydrophobic chromatography [15], it was difficult to extract large amounts of hirudin from natural sources. Hirudin is approximately 7.1 kDa and is composed of 65 amino acids, including a compact N-terminal domain containing three S-S bonds and a C-terminal domain that is disordered in un-complexed hirudin [16,17]. Figure 1 illustrates the interaction of hirudin with napsagatran [13,18].

In contrast to antithrombin III, hirudin inhibited thrombin [13], for treating blood coagulation disorders such as venous thrombosis, coronary thrombosis, and stroke [19]. Recombinant or synthetic hirudin would be of great clinical value because only low yields of hirudin could be extracted from leeches. Bivalirudin is a 20-amino-acid-long synthetic peptide engineered as an analogue of hirudin and has advantages over heparin [20]. The required dosage of bivalirudin was lower, and the anticoagulation mechanism did not depend on intrinsic factors, as was the case of heparin [21]. Bivalirudin was approved by the FDA in December 2000 and was frequently used as an anticoagulant in percutaneous coronary intervention [22,23]. A pharmacokinetic study of recombinant hirudin with chromogenic substrate assay and ELISA demonstrated that its half-life in the plasma in male Sprague Dawley rats was less than 1 h [24].

Additionally, hirudin possessed anti-proliferative activity toward human ovarian cancer cells and tongue squamous cell cancer cells with a synergistic effect with adriamycin *in vitro*[25-27].

### Fibrinolytic enzymes from *Eisenia fetida* (earthworm)

*Eisenia fetida* (earthworm) was used in CM as an antipyretic and anesthetic for treating asthma and hypertension [28]. Ten fibrinolytic enzymes categorized into four groups had been isolated from *Eisenia fetida*[9]. The total number of fibrinolytic enzymes from *Eisenia fetida* was controversial, because the chemical charactersticis, including the full-length amino acid sequences, were not well studied [29]. Seven fibrinolytic enzymes, EFE-a, EFE-b, EFE-c, EFE-d, EFE-e, EFE-f, and EFE-g, were isolated and purified from lyophilized crude powder of earthworm fibrinolytic enzymes by ATKA Purifier, FPLC, and relative pre-packing chromatography columns [30]. These enzymes were crystallized with the hanging-drop vapor-diffusion method [30].

**Figure 2:**
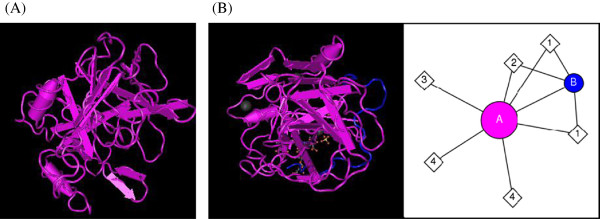
**Molecules and interactions of fibrinolytic enzyme. (A)**, fibrinotic enzyme component A; **(B)**, fibrinotic enzyme component B; A, chain A of fibrinotic enzyme component B; B, chain B of fibrinotic enzyme component B; 1, N-Acetyl-beta-D-glucosaminylamine; 2, alpha-L-Fucose; 3, Mg^2+^; 4, sulfate ion and alpha-D-mannose.

EFE-a, d, and e are approximately 2.4 kDa with isoelectric points (pI) of 3.46, 3.68, 3.62, respectively [30]. EFE-b, c, and g are approximately 3.0 kDa with pI of 3.50, 3.50, 3.46, respectively [30]. EFE-f has a molecular weight of about 2.3 kDa with a pI of 3.94 [30]. Figure 2 shows the interaction of a fibrinolytic enzyme with N-acetyl-beta-D-glucosaminylamine [18,31,32].

Fibrinolytic enzymes exhibited different fibrinolytic activities. EFE-a possessed fibrinolytic activity, with plasminogen-activating activity, which was not present in the other fibrinolytic enzymes [30]. Moreover, earthworm fibrinolytic enzymes exhibit antitumor activity against several hepatoma cell lines *in vitro* and *in vivo*[33], which were potential therapeutic enzymes for hepatomas [34]. After chemically conjugated with human serum albumin fragments, the enzymes lost their antigenicity and acquired resistance to inactivation by protease inhibitors [35]. However, maintenance of enzymatic activity within the body and cleave-site specificity of the enzyme were critical challenges to the practical use of earthworm fibrinolytic enzymes [34].

### An antiepilepsy protein (AEP) from *Mesobuthus martensii* (Chinese scorpion)

Scorpions, especially their tails, were used in CM for the treatment of convulsion and epilepsy [36]. An antiepilepsy protein (AEP) was isolated from the venom of the Chinese scorpion *Mesobuthus martensii* by chromatography, including CM-Sephadex C-50 chromatography, gel filtration on Sephadex G-50, and DEAE-Sephadex A-50 chromatography. The homogeneity of AEP was demonstrated using pH 4.3 polyacrylamide-disc-gel electrophoresis, focusing electrophoresis, and SDS/polyacrylamide-disc-gel electrophoresis [37]. AEP is an 8.3-kDa peptide composed of 61 amino acids, and is derived from an 85-amino-acid precursor. The mature protein contains eight cysteine residues that form four disulfide bonds [38].

**Figure 3:**
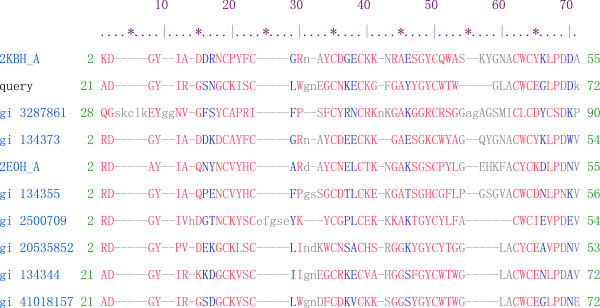
**Alignment of anti-epilepsy peptide conserved domain in Conserved Domain Database (CDD) of NCBI.** The entry used for query was gi|9886761|gb|AAG01571.1|. Aligned residues are in upper case, unaligned residues in lower case, and gaps are displayed as dashes. Red letters indicate highly conserved residues and blue letters indicates less conserved residues Unaligned (lower case) residues are displayed in grey.

AEP exhibited antiepileptic activity by binding to the presynaptic membrane protein synaptosomal-associated protein 25 and the glutamate receptor N-methyl-D-aspartate. Hemorrhagic and toxic activities were not detected in AEP [39]. AEP (28 mg/kg) slightly decreased heart rate in mice without toxicity [37]. The AEP relieved seizures induced by the coriaria lactone without reported side effects in mice [38,40]. The gene encoding AEP was successfully cloned, sequenced, and expressed in a eukaryotic system, providing a rapid method for producing the protein for further mechanistic study [38,39]. Figure 3 shows the alignment of conserved domains in AEP [41].

### Fungal proteins and peptides

#### Lectin and cordymin from *Cordyceps militaris*

*C. militaris* is a less expensive substitute for the mushroom *Cordyceps sinensis* (*Dong Chong Xia Cao*) with different pharmacological properties, including hemagglutinating activity, antifungal properties, and antiproliferative properties [42-44].

A lectin (CML) was isolated and purified from the crude protein extract of *C. militaris* by gel-filtration chromatography on a Sephadex G-75 affinity fetuin-agarose column and dialysis [43]. Purified cordymin was obtained with ion-exchange chromatography of the aqueous extract on SP-Sepharose and Mono S, and gel filtration on Superdex 75 by fast protein liquid chromatography (FPLC) [42]. Another anti-fungal and anti-cancer *C. militaris* protein (CMP), was isolated by anion-exchange chromatography on a DEAE-Sepharose column [44].

CML is approximately 31 kDa and contains the N-terminal amino acid sequence SYDADXQRVXNDKGIXND (the “X” residue could not be identified), which has low homology compared with the lectins from other mushrooms, such as *Marasmius oreades, Laetiporus sulphureus and Polyporus squamosus*[43]. Moreover, the secondary structure of CML contains 27% α-helix, 12% β-sheets, 29% β-turns, and 32% random-coil structures, which is different from the lectins isolated from other mushrooms, such as *Marasmius oreades*, *Laetiporus sulphureus and Polyporus squamosus*[43]. Cordymin is an approximately 10.9-kDa peptide, isolated from *C. militaris*, and its N-terminal sequence is AMAPPYGYRTPDA which is not similar to other peptides in the GeneBank database [42]. Another isolated peptide, *C. militaris* protein (CMP) is a 12-kDa peptide and its pI is 5.1 [44]. Moreover, two parts of the CMP sequence (VSXXGDSGVGGN and NAFNDYTFK) possess more than 70% identity to Rab family GTPases from *Entamoeba histolytica* and exodeoxyribonuclease V alpha chain from *Haemophilus influenza*[44]*.*

CML demonstrated hemagglutinating activity in mouse and rat erythrocytes by specifically binding sialoglycoproteins, and exerted mitogenic activity on mouse splenocytes [43]. Cordymin inhibited not only mycelial growth of numerous fungi, including *Bipolaris maydis*, *Rhizoctonia solani,* and *Mycosphaerella arachidicola*, but also HIV-1 reverse transcriptase and the proliferation of MCF-7 breast cancer cells [42]. CMP could be inhibited by serine protease inhibitors, exhibited an antifungal effect on the growth of *Fusarium oxysporum* at a minimum concentration of 1.6 μM, and had antiproliferative effects on human breast cancer MCF-7 cells (IC_50_ of 9.3 μM) and bladder cancer 5637 cells (IC_50_ of 8.1 μM) [44]. Cordymin prevented osteopenia in diabetic rats by significant up-regulation of bone mineral content and bone mineral density [45].

### Ling Zhi-8 and lectin from *Ganoderma*

*Ganoderma* (*Ling Zhi*) contains a rich content of bioactive constituents and exhibits diverse pharmacological effects, such as anticancer activity, immunomodulatory activity, hypoglycemic action, and hepatoprotection [46]. Moreover, *Ganoderma capense* (Lloyd) Teng was used to tonify the body and improve mental function [47]. Several bioactive peptides have been purified from *Ganoderma*, including Ling Zhi-8 (LZ-8) and *Ganoderma capense* lectin (GCL) [46,48].

LZ-8 (12.4-kDa) was isolated from *Ganoderma lucidum* by Sephadex G-75 column and DEAE-Sephadex A-25 column chromatography, and dialysis, and was characterized by electrophoretic techniques [49,50]. Additionally, an 18-kDA lectin from *Ganoderma capense* (Lloyd) Teng, GCL, was purified by column chromatography on Q-Sepharose and Mono S and gel filtration on a Superdex 75 HR 10/30 column with an AKTA Purifier [49,50].

**Figure 4:**
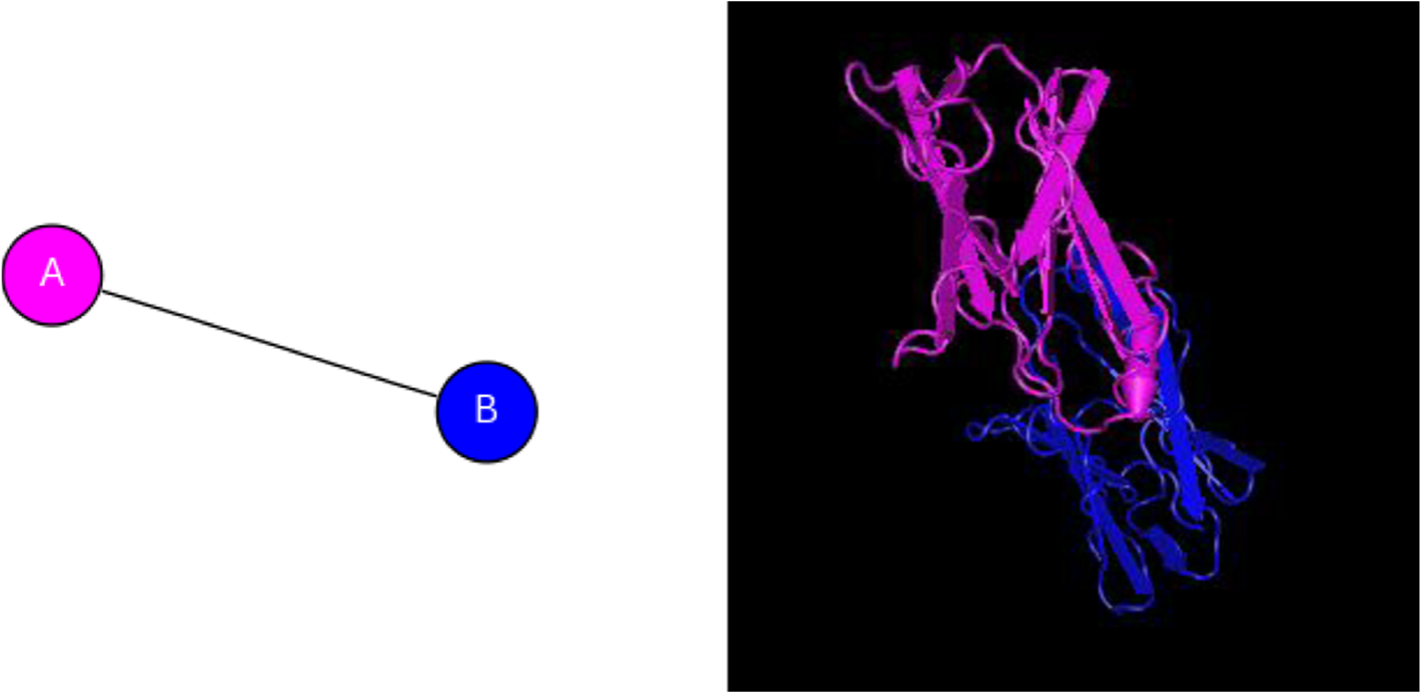
**Crystal structure of LZ-8.A**, Chain A of LZ-8; **B**, Chain B of LZ-8.

LZ-8 is a 12-kDa polypeptide consisting of 110 amino acid residues with an acetylated amino terminus [49,50]. The molecular mass of GCL is 18 kDa, and its N-terminal sequence displays slight similarity to a lectin from *Ganoderma lucidum* and fungal immunomodulatory proteins from *Flammulina velutipes, Volvariella volvacea*[49,50]. The crystal structure of LZ-8 (Figure 4) supplies a basis to study its bioactive function [18,51]. The C-terminal FNIII domain possessed the immunoglobulin-like β-sandwich fold to recognize its target including cytohormones, cell adhesion molecules, cytokine receptors, molecular chaperones and carbohydrate binding domains [51].

Recombinant LZ-8 (rLZ-8) protein induced maturation of human monocyte-derived dendritic cells, which was involved in initiating an antigen-specific T lymphocyte response, by activating the NFκB and MAPK pathways [52]. rLZ-8 also stimulated CD4^+^ and CD8^+^ T cells to secrete IL2 and IFN-γ [53]. GCL exhibited hemagglutinating activity, mitogenic activity toward BALB/c mouse splenocytes, and antiproliferative activity toward leukemia (L1210 and M1) cells and hepatoma (HepG2) cells [54]. rLZ-8 could activate dendritic cells *via* TLR 4 to strength the effectiveness of anti-cancer vaccine [55]. LZ-8 prohibited lung carcinoma cell growth *in vitro* and *in vivo* by regulating p53 [56], and rLZ-8 promoted endoplasmic reticulum stress and the ATF4-CHOP pathway to induce cell death of SGC-7901 human gastric cancer cells *via* autophagy *in vitro*[57]. Moreover, rLZ-8 enhanced the immune response and increased the amount of white blood cells to relieve cyclophosphamide-induced leukopenia [58].

### An immunomodulatory protein (PCP) from *Poria cocos*

*Poria cocos* (*Fu Ling*) reportedly possessed anti-aging, anticancer, and immunomodulatory properties [59]. An immunomodulatory protein (PCP) was isolated and purified from the dried sclerotium of *P. cocos* (Schw.) Wolf with DE-52 cellulose and gel-filtration chromatography, and was characterized by chromatography and electrophoresis [60]. PCP (35.6 kDa) is a disulfide-linked heterodimeric glycoprotein consisting of 14.3 and 21.3 kDa subunits with N- and O-glycosylation [60]. The full-length cDNA sequence of PCP has 807 base pairs and the coding region is 579 base pair encoded 194 amino acids which provides an opportunity to express the recombinant [61].

PCP stimulated mouse peritoneal macrophages (RAW 264.7) by interacting with toll-like receptor 4 and subsequently activating the NFκB signaling pathway [60]. Oral administration of PCP reduced the production of serum total IgG1 and OVA-specific IgG1, as well as up-regulated the serum OVA-specific IgG2a and splenic Th1-related cytokine and down-regulated IL-4 and IgE levels in atopic dermatitis mice [61]. Further studies on PCP will elucidate its modulatory capacity to reveal the pharmaceutical potential and clinical value.

### Herbal proteins and peptides

#### Cyclotides (VTCs) from *Viola tricolor*

*Viola tricolor* was used in CM for *heat* (*re*) dissipation, detoxification, and cough relief [62]. Recently, 14 cytotoxic cyclotides (VTCs) were purified and characterized from dry whole *V. tricolor* by the solvent extraction technique, column chromatography with macroporous resin (D 101), polyamide (100–200 mesh), reverse phase C18 (40–63 μm), and Sephadex LH-20 (25–100 μm), thin layer chromatography, and high performance liquid chromatography [63]. Additionally, TLC was used to detect the products during the procedure [63].

**Figure 5:**
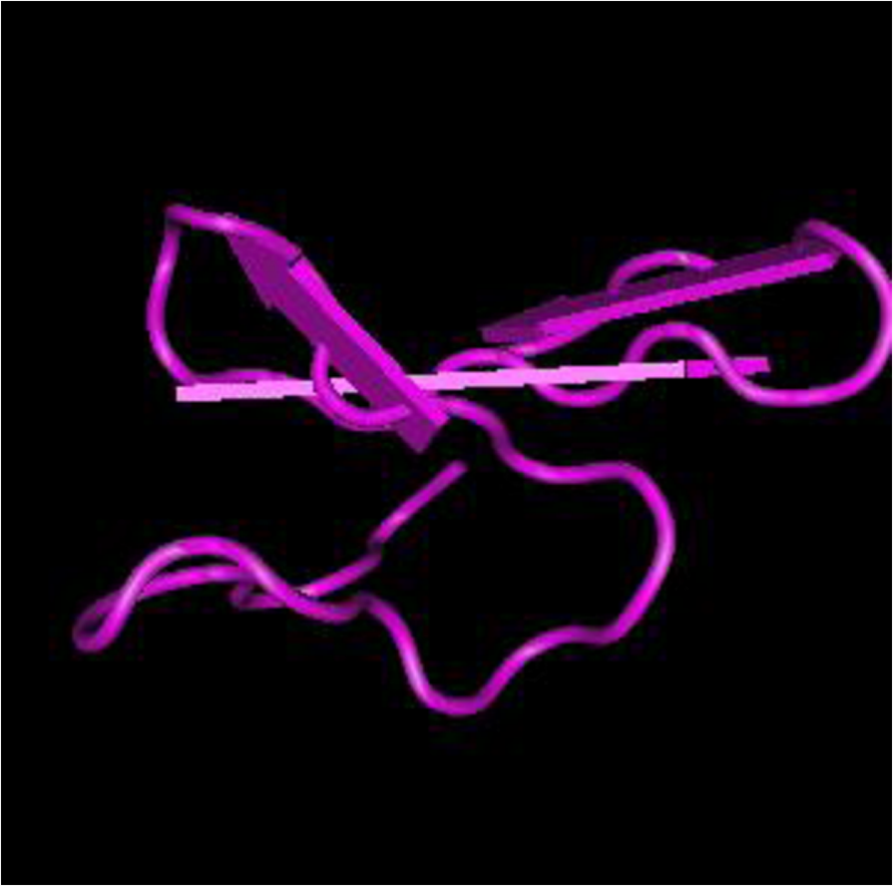
Crystal structure of cyclotides from viola tricolor.

Cyclotides are disulfide-rich proteins that contain a combination of a head-to-tail cyclized backbone and a knotted arrangement of three conserved disulfide bonds that make up a cyclic cystine knot motif [63]. Figure 5 displays the crystal structure of cyclotides from *Viola tricolor*[18,64]. Isolated cyclotides exhibited a cytotoxic effect against human cancer cells U251, MDA-MB-231, A549, DU145, and BEL-7402 [63] and the U-937 GTB and RPMI-8226/s cell lines [65]. Cyclotides exhibited anticancer, anti-HIV, or hemolytic activity *in vitro*[66]. Cyclotides are smaller than most natural proteins, such as nebrodeolysin, and their high stability makes them particularly beneficial to drug design [66].

### Cochinin B, MCoCC-1, and McoCI from *Momordica cochinchinensis* seeds

The seeds of *Momordica cochinchinensis* (*Mubiezhi*) were used as an anti-inflammatory agent to treat hemorrhoids and hemangiomas [67,68]. Several bioactive proteins were isolated and purified from *M. cochinchinensis*, including ribosome-inactivating protein cochinin B, peptide MCoCC-1, and a chymotrypsin inhibitor McoCI by ammonium sulfate precipitation, cation-exchange chromatography on SP Sepharose column, and size-exclusion chromatography on Superdex 75 column with FPLC [69].

The MCoCC peptides were purified from an extract of dried *M. cochinchinensis* seeds by a series of RP-HPLC purifications on Phenomenex C18 columns [68]. Similarly, McoCI was isolated from *M. cochinchinensis* (Lour) seeds by dialysis, chymotrypsin-Sepharose 4B column chromatography and reversed-phase HPLC [70].

**Figure 6:**
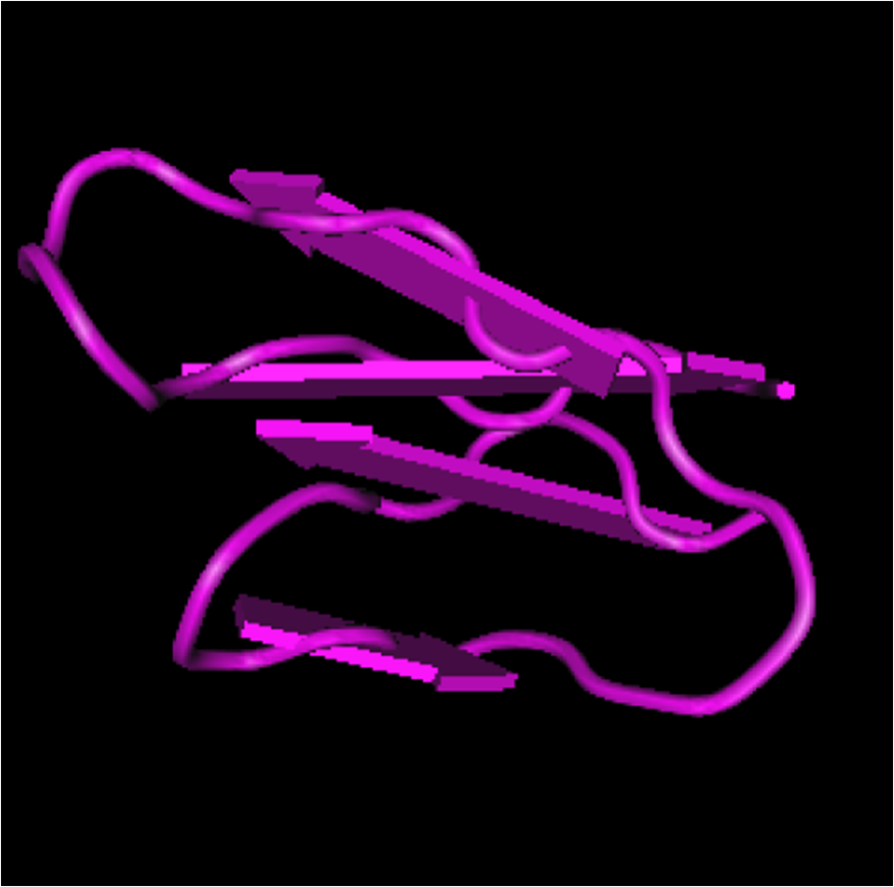
Crystal structure of MCoCC-1 from momordica cochinchinensis.

Cochinin B has a molecular weight of 28 kDa and the N-terminal sequence is DVSFDMSTASTESYKKFIAD, which displays 45–60% identity to other type I RIPs in the Cucurbitaceae family recorded in GenBank [69]. MCoCC-1 and MCoCC-2, which are approximately 7.5 k Da, have partial sequences of 33 and 32 amino acid residues, respectively, which differ only in two residues. They are also Cys-rich peptides with a cystine knot motif [68]. Figure 6 shows the crystal structure of MCoCC-1 from *M. cochinchinensis*[18,68].

Cochinin B, a ribosome-inactivating protein, exhibited strong anticancer activity against human cervical epithelial carcinoma (HeLa), human embryonic kidney (HEK293), and human small cell lung cancer (NCI-H187) cell lines [69]. MCoCC-1, exhibited cytotoxic effects against human melanoma cell line MM96L and was nonhemolytic [68]. McoCI, a 7.5-kDa antioxidative and immunomodulatory potato I family chymotrypsin inhibitor, reversed oxidative injury in *t*-BHP-challenged rat hepatocytes *via* the antioxidative activity of glutathione-S-transferase and superoxide dismutase [71]. It stimulated the proliferation of mouse splenocytes, splenic lymphocytes, bone marrow cells, and macrophages, and inhibited H_2_O_2_ production by macrophages and neutrophils [67].

### Lectin CM-1 and ACML-55 from *Viscum album*

*Viscum album* (Mistletoe) was used as a complementary anticancer medicinal herb [72]. A mistletoe lectin-I, designated as CM-1, was isolated from the leaves of *V. album* and purified by affinity chromatography and cation-exchange chromatography [73]. ACML-55 was isolated by solvent extraction and purified by CM-Sepharose column chromatography [74].

CM-1, a 55-kDa lectin, down-regulated micro-RNA miR-135a and miR-b expression, leading to up-regulation of adenomatous polyposis coli gene expression and attenuation of the Wnt pathway in CLY colon cancer cells and HT-29 colorectal cancer cells, both *in vivo* and *in vitro*[73]. ACML-55 enhanced both antigen-specific activation and proliferation of CD4^+^ and CD8^+^ T cells and a number of tumor antigen-specific CD8^+^ T cells in colon cancer cell line CT 26 in BALB/c mice [74]. The number of natural killer cells and gamma-delta T cells was also elevated, indicating that ACML-55 modulated innate and adaptive immune responses [74]. The lectin from *V. album* activated autophagy to promote the proliferation of placenta-derived mesenchymal stem cells *via* upregulation of type II LC3 and downregulation of phosphorylated mTOR [75].

### A cholesterol-lowering protein from *Senna obtusifolia* seed

*Senna obtusifolia* seeds (*Juemingzi*) were used in CM to treat hyperlipidemia and hypertension, and to remove *liver heat* (*gan re*) [76,77]. Moreover, a novel cholesterol-lowering protein was isolated and purified from *S obtusifolia* seeds by gel-filtration and ion-exchange chromatography [78].

This cholesterol-lowering protein is a single protein with a molecular weight of 19.7 kDa and a pI of 4.8 [78]. N-terminal amino acid sequence of this peptide, IPYISASFPLNIEFLPSE, has no homology with any other protein sequences in the GeneBank [78]. Its secondary structure has 12.5% α-helix, 55.6% β-sheet, and 31.9% random coil [78].

This cholesterol-lowering protein inhibited cholesterol biosynthesis in Chinese hamster oocytes [78]. Statins were commonly used as HMG-CoA reductase inhibitors to reduce the blood cholesterol level. However, they had adverse effects, mainly on muscles but occasionally on nonmuscle tissue [79]. Thus, the hyperlipidemic mechanism and safety of the novel protein from *Juemingzi* must be thoroughly evaluated.

### Lectin (NTL) from *Narcissus tazetta* var. *chinensis*

*Narcissus tazetta* var. *chinensis* was an anticancer medicinal herb. *N. tazetta* lectin, (NTL) was isolated and purified by various chromatographies, including ion- exchange chromatography on diethylaminoethyl (DEAE)-cellulose, affinity chromatography on mannose-agarose, and FPLC-gel filtration on Superose 12 [80].

NTL has a molecular weight of 26 kDa, and is probably a dimer with two identical subunits. NTL protein, predicted by cDNA sequence, contains a mature polypeptide and a C-terminal peptide extension [80]. Moreover, NTL primary polypeptide contains three subdomains, each with a conserved mannose-binding site. NTL is 60–80% identical to other known monocot mannose-binding lectins [80].

NTL inhibited plaque formation from human syncytial virus (RSV) and antiviral properties against influenza A and B [80]. NTL exhibited an immunopotentiating effect, similar to that of LZ-8, by inducing gene expression of IL-1β, TNF-α, and nitric oxide synthase in splenocytes and macrophages *in vivo*[81].

### Smilaxin from *Smilax glabra* rhizomes

*Smilax glabra* rhizomes (*Tufuling*) had antipyretic, detoxifying, and diuretic effects and were used in the treatment of brucellosis, syphilis, furunculosis, eczema, dermatitis, nephritis, cystitis, and mercury and silver poisoning [82]. Smilaxin was isolated from fresh *S. glabra* by successive column chromatography on DEAE-cellulose, CM-cellulose, Con A-Sepharose, and Mono S, and FPLC-gel filtration on Superdex 75 [83]. Smilaxin is a 30-kDa protein, and the N-terminal sequence of smilaxin is homologous to few proteins [83].

Smilaxin exhibited immunostimulatory, antiproliferative, and HIV-1-reverse transcriptase inhibitory activities [83]. Smilaxin specifically stimulated the uptake of [methyl-^3^H]thymidine in murine splenocytes, peritoneal macrophages, and bone marrow cells, but not in MBL2 or PU5 tumor cells [83]. It also attenuated the activity of HIV-1 reverse transcriptase with an IC50 of 5.6 μM [83].

### Ginkbilobin from *Ginkgo biloba* seeds

*Ginkgo biloba* seeds were used in CM for relieving cough and asthma, reducing phlegm, and leukorrhea, and treating incontinence [84]. Ginkbilobin was isolated and purified from *G. biloba* seeds by dialysis, DEAE-cellulose column chromatography, Affi-gel blue gel chromatography, ion-exchange chromatography, and FPLC-gel filtration [85]. Purified Ginkbilobin-2 was obtained by Sephadex G-50 column and ion-exchange chromatography [86].

**Figure 7:**
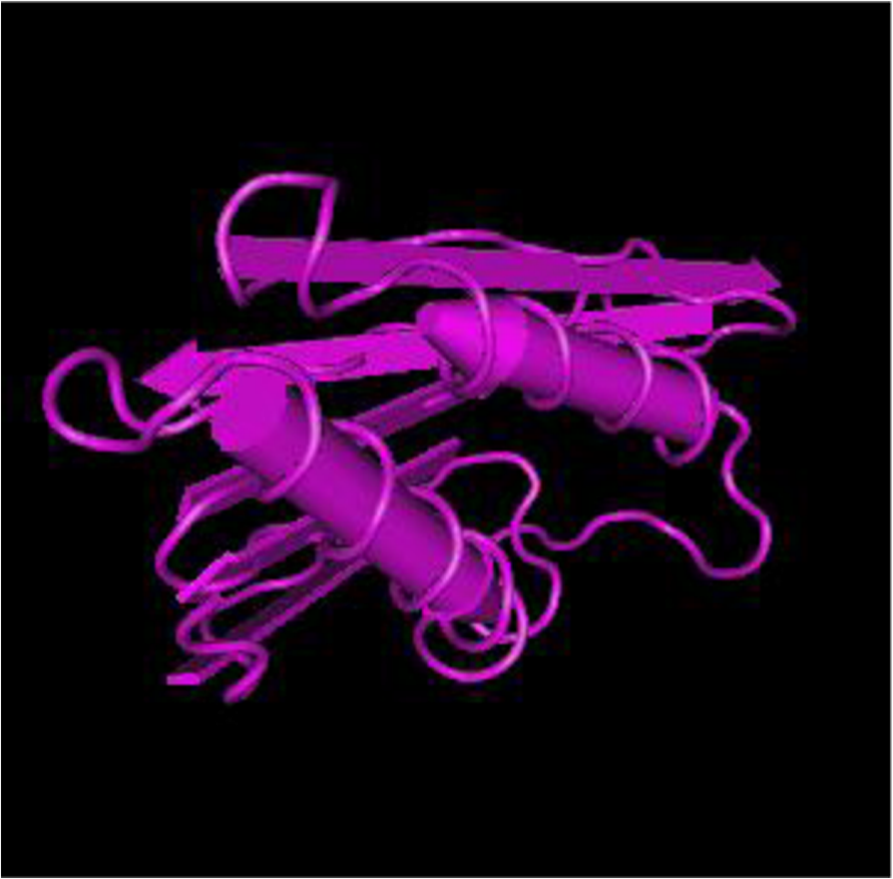
Crystal structure of Ginkbilobin-2 of Ginkgo biloba seeds.

Both ginkbilobin and ginkbilobin-2, are ~13-kDa proteins with 108 amino acids, which is the product of a pro-peptide containing 134 amino acids and a potential signal peptide (26 residues) with approximately 85% identity to embryo-abundant proteins from *Picea abies* and *Picea glauca*[87]. The differences in amino acid sequence between ginkbilobin and ginkbilobin-2 are His10/Cys, Ala17/Ser, Ala19/Ser, Ala36/Thr, and Ala39/Ser. Ginkbilobin has two α-helices and a five-stranded β-sheet, which form a compact single-domain architecture with an α + β-fold. The crystal structure of Ginkbilobin-2 was shown in Figure 7[18,88]. The positively charged surface of ginkbilobin-2 might react with the negatively charged surface of fungal cells to display its antifungal activity [87].

Ginkbilobin exhibited antifungal activity against *Botrytis cinerea*, *Mycosphaerella arachidicola*, *Fusarium oxysporum*, *Rhizoctonia solani*, and *Coprinus comatus* and antibacterial activity against *Staphylococcus aureus*, *Pseudomonas aeruginosa*, and *Escherichia coli*[85]. In addition, it inhibited HIV-1 reverse transcriptase and proliferation of murine splenocytes [85]. The pharmacology of ginkbilobin-2 should be studied in the future.

### Dioscorin from *Dioscorea batatas*

Tubers from the *Dioscorea* genus were widely used in CM to relieve the menopausal syndrome [89]. A tuber storage protein from *Dioscorea batatas*, dioscorin, was isolated and purified by ammonium sulfate fractionation, DE-52 ion-exchange chromatography, and Sephadex G-75 column chromatography [90].

*Dioscorea batatas* dioscorin, a 32-kDa protein, protect against airway damage due to the trypsin activity of dust mites by reversing the expression of tight junction proteins [90]. It prevented hydrogen peroxide-induced oxidative damage *via* down regulating IL-8 secretion and adhesion molecule expressions, and possibly activating IκB in A549 human airway epithelial cells [91,92]. Dioscorin also scavenged 1,1-diphenyl-2-picrylhydrazyl radicals and capture hydroxyl radicals, indicating it had antioxidant properties [93]. The immunomodulatory properties of dioscorin included stimulation of cytokine production and nitric oxide production, RAW 264.7 phagocytosis of *E. coli*, and mitogenic effects on mouse splenocytes [94]. These properties were indicated by *in vivo* data from dioscorin-treated BALB/c mice [95].

### Trichosanthin from *Trichosanthes kirilowii*

The root of *Trichosanthes kirilowii* was used in CM for treating *lung heat* (*fei re*) and inflammation [96]. A trichosanthin (TCS) was isolated from *T. kirilowii* root tubers by acetone fractionation and CM-Sepharose ion-exchange chromatography [97]. Recently, Arijit Mondal extracted and purified TCS with three-phase partitioning (TPP), which was cost-effective and environmental friendly [98].

**Figure 8:**
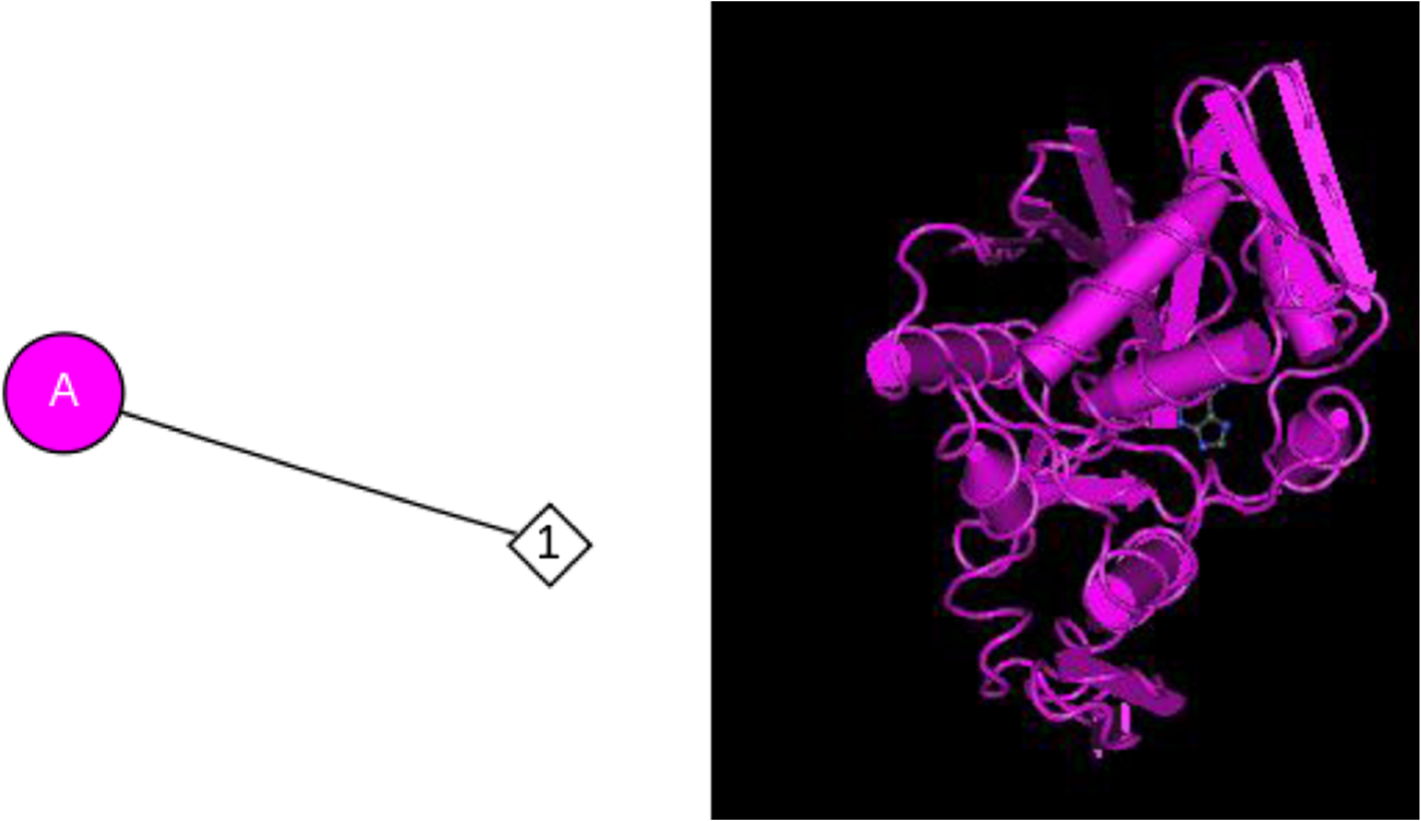
**Crystal structure of the complex of trichosanthin of Trichosanthes kirilowii with adenine.** A stands for chain of trichosanthin; 1 represents adenine.

TCS is a single-chain 24-kDa protein with 247 amino acid residues including a 23-amino acid N-terminal signal peptide and a 19-amino acid C-terminal pro-peptide [99-101]. TCS contains 12.2% α-helices, 16.3%, β-sheets, 51.4% turns, and 20.1% random curls. TCS is a type I ribosome-inactivating protein, which inactivated ribosomes by site-specific cleavage of the single N–C glycosidic bond and prevented the elongation factor from binding to the 60S ribosomal subunit, resulting in arrest of protein synthesis [102]. The mature protein is homologous to other ribosome-inactivating proteins (RIPs). The peptide SDDDMGFGLFD is related to the conserved C-terminal elongation factor binding zone of the ribosomal P protein and is similar to ricin A [103]. Additionally, the crystal structure of the complex of trichosanthin with adenine was shown in Figure 8[18,104].

Isoforms of TCS, α-, β- and γ-TCS, with similar biochemical activities have been reported [105]. Also, Wang *et al.*[106,107] constructed two mutants of TCS, RL28-29CG and FYY163-165CSA in *E.coli*. TCS lowered serum HIV-1 p24 antigen levels and increased CD4^+^ T cell number in HIV-1 infected patients [108,109]. TCS also inhibited hepatitis B virus and tumor growth [110,111]. Additionally, TCS exhibited anti-cancer effects. TCS promoted breast cell cancer cell line apoptosis *in vitro* and in nude mice [112], and inhibited tumour migration *in vitro* and angiogenesis in the aortic ring model without side effects [113]. TCS inhibited lung cancer proliferation and induced apoptosis, and also enhanced the immunoreaction by increasing the expression and interaction of tumor suppressor in lung cancer 1 (TSLC1) and class I-restricted T cell-associated molecule (CRTAM) [114]. TCS suppressed nasopharyngeal carcinomas *via* inhibiting Notch signaling and proliferation *in vitro*[115], as well as induced cell death and inhibited telomerase activity in nude mice [116]. Moreover, TCS possessed anti-HSV-1 property in human epithelial carcinoma cell line HEp-2 *via* type II apoptotic signaling after infection [117], and inhibiting the activation of NF-κB and inducing p53-dependent apoptosis [118]. TCS was used to successfully cure 85% of 140 cases of ectopic pregnancy with higher beta-human chorionic gonadotropin [119].

TCS has limited clinical applications due to its major adverse effects, including short plasma half-life, immunogenicity, and neurotoxicity [120,121]. However, TCS exhibited less cytotoxicity than type II ribosome-inactivating proteins, including abrin, ricin, *etc.*[120]. Researchers employing recent advances in drug delivery technology have reported that site-directed PEGylation of trichosanthin could decrease immunogenicity, and prolong plasma half-life [7].

### Future development

Research of bioactive proteins and peptides in CM is still in the early stage of development. A number of them have been isolated and characterized, but only a few are commercially available as pharmaceuticals, such as Hirudin. Advances in technologies would facilitate development of protein pharmaceuticals in CM. Approaches to discovery of the action mechanisms of proteins from CM would be crucial to translate CM protein in synergistic proportions into pharmaceutical for clinical use.

## Conclusion

The bioactive proteins and peptides isolated from CM have therapeutic potentials but further study and pharmaceutical development would be necessary for clinical use.

## Abbreviations

CINAHL Plus: Cumulative Index to Nursing & Allied Health Plus; CNKI: China National Knowledge Infrastructure; CQVIP: Chongqing VIP Information; CJFD: China Academic Journals Full-text Database; WHO: World Health Organization; MMDB: Molecular Modeling Database; CDD: Conserved Domain Database; NCBI: National Center for Biotechnology Information; AEP: anti-epilepsy protein; CM: Chinese medicine; CML: *Cordyceps militaris* Lectin; CMP: *Cordyceps militaris* protease; EFE: earthworm fibrinolytic enzyme; FDA: Food and Drug Administration; FPLC: Fast protein liquid chromatography; GCL: *Ganoderma capense* lectin; LZ-8: Ling Zhi-8 protein; NTL: *Narcissus tazetta* lectin; PCP: *Poria cocos* immunomodulatory protein; PEG: polyethylene glycol; TCS: trichosanthin; VTCs: *Viola tricolor* cyclotides.

## Competing interests

The authors declare that they have no competing interests.

## Authors’ contributions

SCWS, YBZ, ZJZ, and YT designed and conceived the study. SCWS, RNSW, WKL, TBN and PCS, select and organize the contents of manuscript. KLW, LZ, HPC, and JL wrote the manuscript. TBN, PCLK and YML provided the constructive comments and re-wrote parts of manuscript. All authors read and approved the final version of the manuscript.
